# SciRAPepi Tool: Optimization of the SciRAP Tool for Evaluating the Reliability and Relevance of Observational Epidemiological Studies for Hazard and Risk Assessment of Chemicals

**DOI:** 10.1021/acs.est.5c11558

**Published:** 2026-01-16

**Authors:** Henrieta Hlisníková, Anna Beronius

**Affiliations:** Institute of Environmental Medicine, Karolinska Institute, Nobels väg 13, 17177 Stockholm, Sweden

**Keywords:** SciRAP, SciRAPepi tool, hazard and risk assessment of chemicals, evidence appraisal tools, observational epidemiological studies, reliability, relevance

## Abstract

The Science in Risk Assessment and Policy (SciRAP) platform has been developed to support the structured and transparent evaluation of data in hazard and risk assessment of chemicals. This work aimed to develop a SciRAP tool for the evaluation of the reliability and relevance of observational epidemiological studies (SciRAPepi tool), including cross-sectional, classical case-control, nested case-control, and cohort studies. A first version of the SciRAPepi tool was created, and an expert testing round was conducted to assess its scientific soundness and user-friendliness. Thirty-seven epidemiologists and risk assessors took part in the testing using the tool to evaluate four epidemiological studies representing the main epidemiological study designs. The majority of experts considered the criteria appropriate, and overall evaluations indicated good consistency across most criteria. Based on the testing results, the tool was further developed and refined. Together with the SciRAPepi tool, guidance and instructions on its use were developed, along with a reporting checklist to support researchers in reporting their studies. The SciRAPepi tool intends to enhance the use of reliable epidemiological data in the hazard and risk assessment of chemicals and other scientific assessments, offering a broader range of options for evaluating these data for epidemiologists and risk assessors.

## Graphical abstract

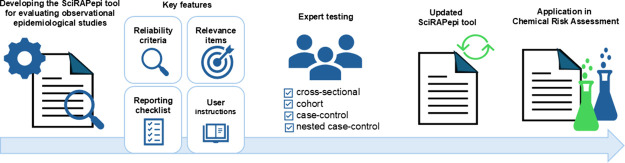

## 1 Introduction

As the number of chemicals in use continues to grow, the need for robust chemical risk assessment becomes increasingly important to protect human health and the environment, promote safety in occupational and consumer products,^1^ build public trust,^2^ and support sustainable development.^3^ Epidemiological data, when available, hold high relevance for human health risk assessment of chemicals. For instance, epidemiological data have been pivotal in the assessments from the European Food Safety Authority (EFSA), such as per- and polyfluoroalkyl substances (PFAS)^4^ and cadmium,^5^ where human studies were used as key evidence in determining health risks.

However, only sufficiently reliable data that are relevant to a specific risk assessment question should be used. Epidemiological studies are subject to several inherent limitations, including confounding factors and limited sample sizes^6^ that hinder the ability to clearly establish causal links between exposure and outcome^7^; challenges in accurate exposure assessment, particularly when relying on self-reported data^8^; and restricted generalizability when specific populations are studied.^9^ These issues can affect the interpretation of associations and reduce the relevance of findings for broader risk assessment applications.

Moreover, there are no Organisation for Economic Co-operation and Development (OECD) test guidelines defining standard methods for epidemiological research in chemical risk assessment, and human data are not routinely required in regulatory submissions outside of pharmaceuticals.^10^ The OECD has only very recently published a Guidance Document on the Generation, Reporting and Use of Research Data for Regulatory Assessments, which provides guidance on the interpretation of observational epidemiological studies, particularly with respect to study reliability, with a focus on participant selection, confounding factors, exposure assessment, and study sensitivity.^11^ Consequently, risk assessment commonly relies on animal studies, where controlled experimental conditions offer clearer causal interpretation.^12^

In chemical risk assessment, reliability reflects the inherent quality of a study and the confidence in its findings, whereas relevance refers to the degree to which the data address the assessment question.^13,14^ These concepts closely align with the terms internal validity and external validity used in systematic reviews of epidemiological evidence.^15,16^ Although terminology differs between fields, they fundamentally address similar principles for study evaluation. More detailed definitions are provided in Table 1.

**Table 1:** Definitions of Terminology Used in Risk Assessment and Systematic Review

**terminology**	**area of use**	**definition**
reliability	risk assessment	the inherent quality of a study and the confidence in the findings as well as the reproducibility of findings between experiments^13,14^
relevance		the extent to which data and tests are appropriate for a particular hazard identification or risk characterization^13,14^
internal validity	systematic review	the extent to which the observed results represent the truth in the population that is studied and, thus, are not due to methodological errors^15,16^
external validity		the extent to which the results of a study are generalizable to the population that the sample is thought to represent^15,16^

Structured and harmonized approaches to assessing the reliability and relevance of epidemiological studies become imperative to enable their utilization in health risk assessment of chemical substances.^12^ Several risk of bias (RoB) tools have been developed in the last few decades to facilitate the evaluation of the quality of observational epidemiological studies for systematic reviews and weight of evidence (WoE) assessments.^17^ Some examples are The Risk of Bias in Non-Randomized Studies – of Exposure (ROBINS-E),^18^ the Newcastle-Ottawa Scale (NOS),^19^ Quality Assessment Tools for Observational Cohort and Cross-Sectional Studies,^20^ OHAT RoB Rating Tool for Human and Animal Studies.^21^ These tools primarily address reliability/internal validity, focusing on the risk of bias in study design, conduct, and reporting. Tools such as Strengthening the Reporting of Observational Studies in Epidemiology (STROBE) provide reporting guidance.^22^

The Science in Risk Assessment and Policy (SciRAP) web-based platform (http://www.scirap.org) has been developed to aid structured assessment of reliability and relevance of in vivo and in vitro toxicity as well as ecotoxicity data for use in chemical hazard and risk assessment, and particularly to facilitate and promote the use of research data in regulatory assessments. The SciRAP tools offer a systematic approach to evaluate nonstandard in vivo, in vitro, and ecotoxicity studies, promoting transparency, consistency, and robustness in the assessment of evidence.^13,23–25^ However, no analogous tool within the SciRAP platform exists for human epidemiological data, which limits the harmonization of evidence evaluation across study types.

This study aimed to develop a SciRAP tool for evaluating the reliability and relevance of epidemiological data for use in assessments of health hazards and risks of chemicals, the “SciRAPepi”. The tool covers four common study designs (cross-sectional, classical case-control, nested case-control, and cohort). SciRAPepi does not aim to replace existing approaches but to complement them by extending the SciRAP platform to epidemiological studies, enabling assessors to use one coherent system across in vitro, in vivo, ecotoxicology, and now epidemiological studies. It is important to highlight that the primary aim of the study was not to conduct validation of the SciRAPepi tool, or to quantitatively measure factors like inter-rater or intrarater reliability. The second aim of our study was to develop a reporting checklist to support transparent reporting that facilitates the use of human data in regulatory decision-making.

## 2 Materials and Methods

### 2.1 Development of SciRAPepi Tool (Version 1.0)

The development of the SciRAPepi tool (Figure 1) was based on the previously developed SciRAP tools, which were designed for assessing the in vitro,^25^ in vivo,^13^ and ecotoxicity studies.^26^

**Figure 1:**
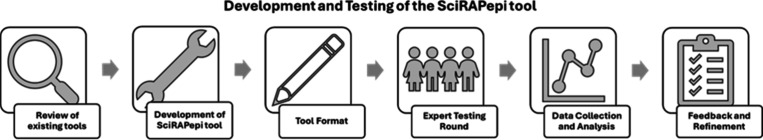
Development and testing of the SciRAPepi tool.

To create specific criteria for the evaluation of data from epidemiological studies, several assessment tools for such studies were reviewed, including:

- STROBE: This tool provides guidelines for reporting of observational studies.^22^
- Handbook for Conducting a Literature-Based Health Assessment Using OHAT Approach for Systematic Review and Evidence Integration: This handbook offers guidance for conducting health assessments using systematic review approaches.^21^
- NIH quality assessment tool: This tool helps evaluate the quality of epidemiological studies.^20^
- NOS: A widely used tool for assessing the quality of observational studies, specifically cohort and classical case-control designs.^19^

These criteria were designed specifically for observational epidemiological studies, also with consideration of assessment tools mentioned above, concerning study participants and study design, exposure assessment, outcome assessment, statistical analysis and data processing, and ethics and competing interests.

### 2.2 Expert Test Round

#### 2.2.1 Expert Test Round Procedure

The SciRAPepi tool Version 1.0 was evaluated in an expert test round from October 2023 to February 2024, with the aim of assessing its practical use among assessors from various sectors and geographical regions. Experts received instructions, four studies, corresponding Excel workbooks, and a survey link via email. They were asked to assess each study’s reliability and relevance using the tool and submit their results by December 2023, with an optional extension to February 2024. Each expert completed two tasks^1^: evaluating the studies using SciRAPepi (Tables S1–S3 list the reliability and relevance items used during the test round), and^2^ responding to a survey (see Table S4) to provide feedback on the tool’s criteria, guidance, and overall usability.

In addition to the evaluation, experts’ affiliation, country of residence, and years of experience in epidemiology/risk assessment were collected. To ensure compliance with data protection regulations, all experts gave informed consent for the collection and storage of their personal information in accordance with the European General Data Protection Regulation (GDPR) and Swedish rules concerning the archiving of research data. Participation in the expert test round was voluntary, and experts had the option to withdraw at any time without providing a reason.

#### 2.2.2 Selection of Experts in Epidemiology and Risk Assessment

From September to November 2023, experts specializing in epidemiology and chemical risk assessment from regulatory authorities, academia, industry, and consultancy were invited to take part in the test round. The selection of experts was not conducted randomly. Invitations were sent via email to experts within our network of contacts in Europe, North America, South America, Australia, and Asia. Experts we contacted were encouraged to distribute the invitation in their own networks. Prior to agreeing to participate, the experts were provided with information on how their personal data would be handled and asked to give their informed consent.

#### 2.2.3 Selection and Evaluation of the Test Studies

The experts were asked to evaluate the reliability and relevance of the same four epidemiological studies using the SciRAPepi tool version 1.0:

- Study 1: Association between exposure to formaldehyde and respiratory disease (Cross-sectional study).
- Study 2: Association between exposure to phthalates and hypothyroidism (Classical case-control study).
- Study 3: Association between exposure to heavy metals and *diabetes mellitus* (Nested case-control study).
- Study 4: Association between exposure to mercury and puberty onset (Cohort study).

The selection of studies was conducted based on the following criteria: the studies had to be openly accessible and recently published (<5 years). The primary focus of the studies should be on evaluating the relationship between chemical exposure and human health effects. The studies were not excessively complex and involved exposure to a single substance without mixtures, while investigating health effects within a specific body system (e.g., endocrine system, respiratory, or reproductive system) individually. Four studies with diverse study designs (cross-sectional, classical case-control, nested case-control, and cohort studies) were selected to examine exposure to different chemicals and a range of health effects. It was also intended to include studies with varying levels of reporting quality (RQ), with at least one well-documented study and one study with relatively less comprehensive reporting.

### 2.3 Data Analysis

#### 2.3.1 Study Evaluation

A custom macro was created using Visual Basic for Applications in Microsoft Excel to facilitate the import and formatting of specific data from all Excel workbooks submitted by experts for each study into a single Excel sheet. Additionally, Microsoft Excel was utilized to conduct qualitative and quantitative analyses and comparisons of experts evaluating the reliability and relevance criteria and items for each study individually and across multiple studies.

Decision rules were established to prioritize reliability criteria for potential refinement by assessing the variability in expert evaluations when using the SciRAPepi tool (Figure 2). A criterion required no further refinement if at least 50% of experts assigned it to the same rating category. Conversely, if the distribution of expert ratings across categories was ≤49.99%, further evaluation of whether the criterion needed refinement was warranted. Specifically, if inconsistencies in the evaluation of criteria were observed across all four studies, these criteria should be re-evaluated. However, if only one study exhibited inconsistencies in evaluating specific criteria, no further refinement was necessary. In contrast, if at least two studies displayed inconsistent evaluations, the criterion required further refinement. Table S5 provides detailed information regarding the decision process for further refinement.

**Figure 2:**
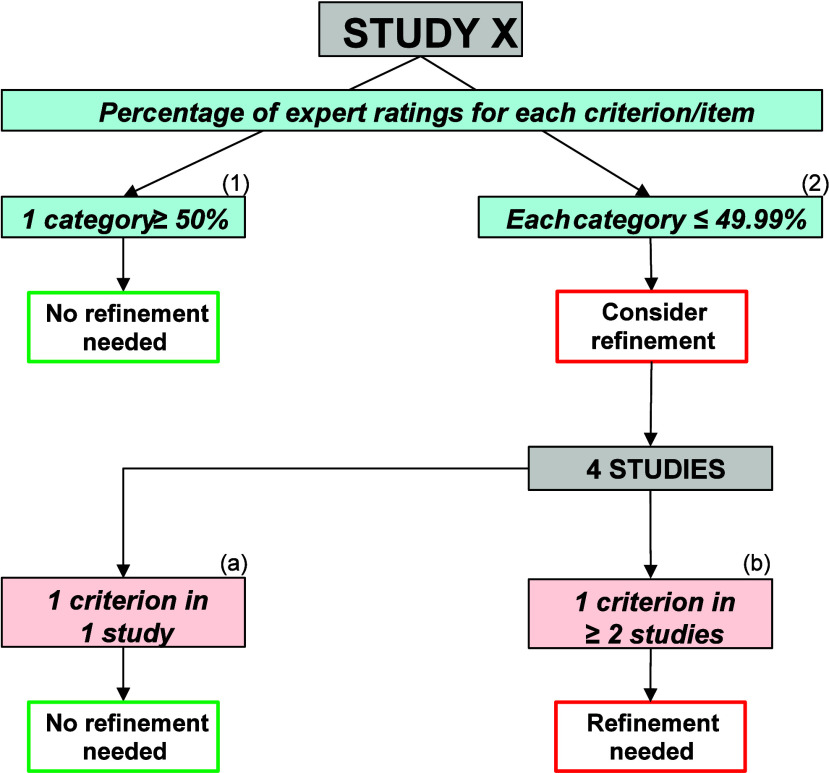
Decision rules for evaluating variability in expert ratings for reliability criteria and relevance items, considering the percentage of expert ratings for each category. Decision rules are applied to prioritize criteria and items for further improvement. Decision rules: (1) ≥50% of experts have allocated the criterion/item to the same rating category. There is no need for improving the criteria/item under consideration. (2) The criteria/item has been allocated by experts to three or more rating categories. If the percentage of expert ratings for each category is ≤49.99%, then the need for improvement should be further evaluated as follows: All four studies should be considered for final evaluation of a specific criterion whose evaluations were inconsistent among experts. If only one study showed inconsistent evaluations of a specific criterion, there is no need for further improvement (a). If at least two studies showed inconsistent evaluations of a specific criterion, there is a need for improvement (b).

#### 2.3.2 Online Survey

The Survey & Report tool (https://www.artologik.com/en/SurveyAndReport.aspx) exports survey results to an Excel file, which was then used to analyze the survey data. Predominantly, closed-ended questions were used in the survey. The subsequent qualitative analysis involved extracting and analyzing information related to experts’ demographics and feedback on specific criteria and tool use. Additionally, the qualitative analysis involved extracting data from open-ended questions regarding comments and suggestions for improving the SciRAPepi tool. These comments were valuable for further tool refinement.

## 3 Results

### 3.1 Results of the SciRAPepi Tool Testing

A total of 37 experts participated in the testing of the SciRAPepi tool version 1.0. Detailed information about the demographics of the experts is presented in anonymised form in Table 2. Specifically, 35 experts evaluated Study 1, 37 evaluated Study 2, 35 evaluated Study 3, and 36 evaluated Study 4. The number of experts completing the online survey also varied, with 30 experts categorizing Study 1 and Study 3, and 31 experts categorizing Study 2 and Study 4 into different reliability categories. The differences in the number of experts evaluating each study reflect the time demands associated with assessing four studies of varying designs. Some experts were unable to complete all evaluations due to time constraints. To maximize participation, we allowed experts to contribute to a subset of the studies, which were assigned to them. A similar challenge occurred with the online survey, where not all experts provided responses.

**Table 2:** Demographics of Participants in the Expert Round Test of the SciRAPepi Tool (Version 1.0)

**demographics of participants**	**no. of participants in the test round (%)**
total number of participants	37 (100)^a^
number of participants that completed the survey	32 (86.49)
Geographical Area^a^	
Europe	28 (75.68)
USA	6 (16.22)
out the Europe and USA	3 (8.10)
Current Affiliation^a^	
academia	17 (45.95)
authority and government	17 (45.95)
industry and consultancy	3 (8.10)
Years of Experience in Epidemiology	
none	5 (13.51)
≤1	0 (0)
1–5	8 (21.65)
6–10	10 (27.03)
11–15	5 (13.51)
>15	4 (10.81)
Years of Experience in Risk Assessment of Chemicals	
none	3 (8.11)
≤1	5 (13.51)
1–5	10 (27.03)
6–10	5 (13.51)
11–15	5 (13.51)
>15	4 (10.81)

a 37 participants entered the SciRAPepi tool testing, but 5 participants did not participate in the online survey, where details regarding demographics and expertise were collected. However, we could extrapolate the affiliation and geographical area details based on contact information.

Figure 3A–D illustrate how each reliability criterion and relevance item was assessed by each expert for each study, with the tool version 1.0 criteria and items detailed in Tables S1–S3.

**Figure 3:**
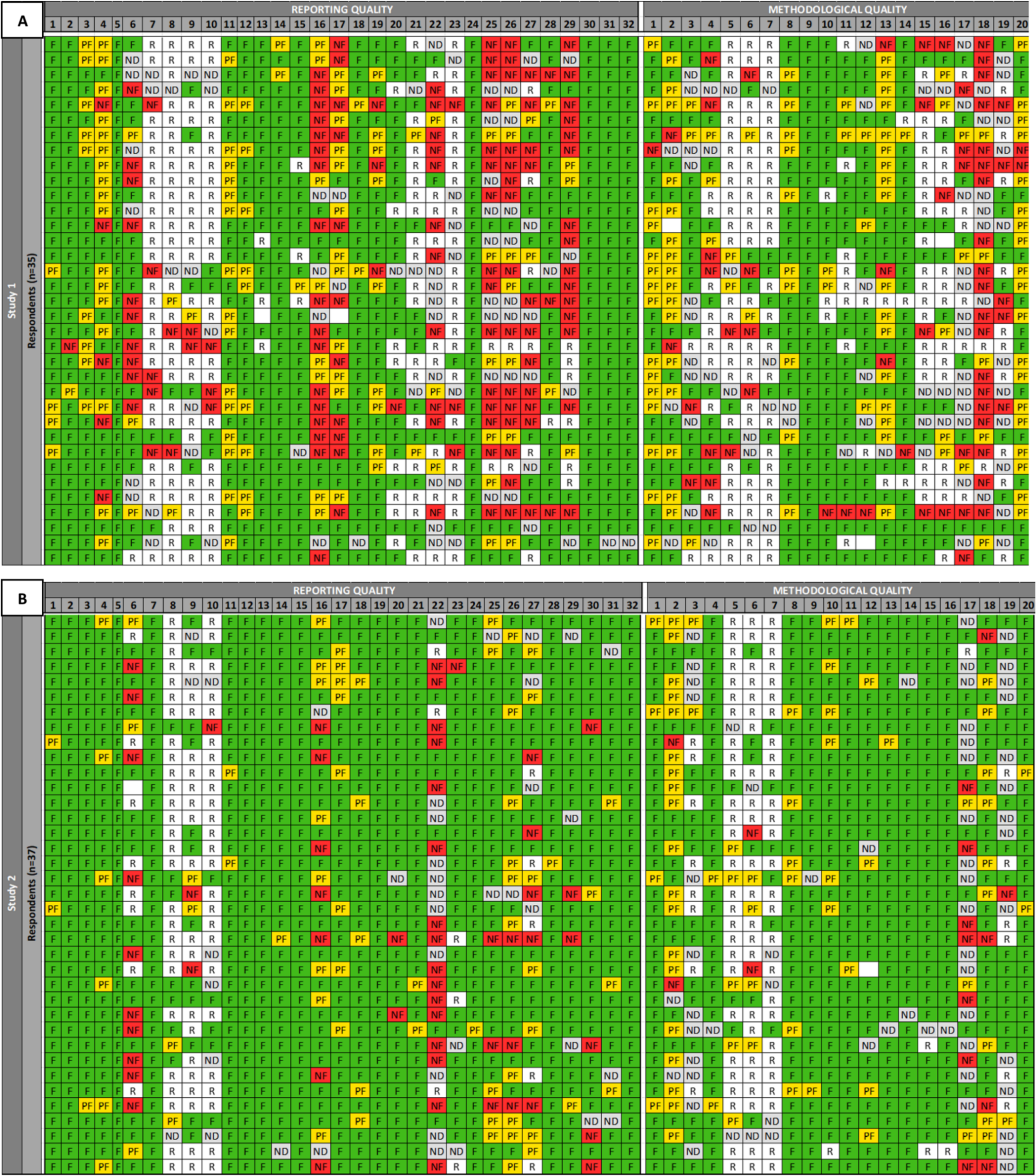

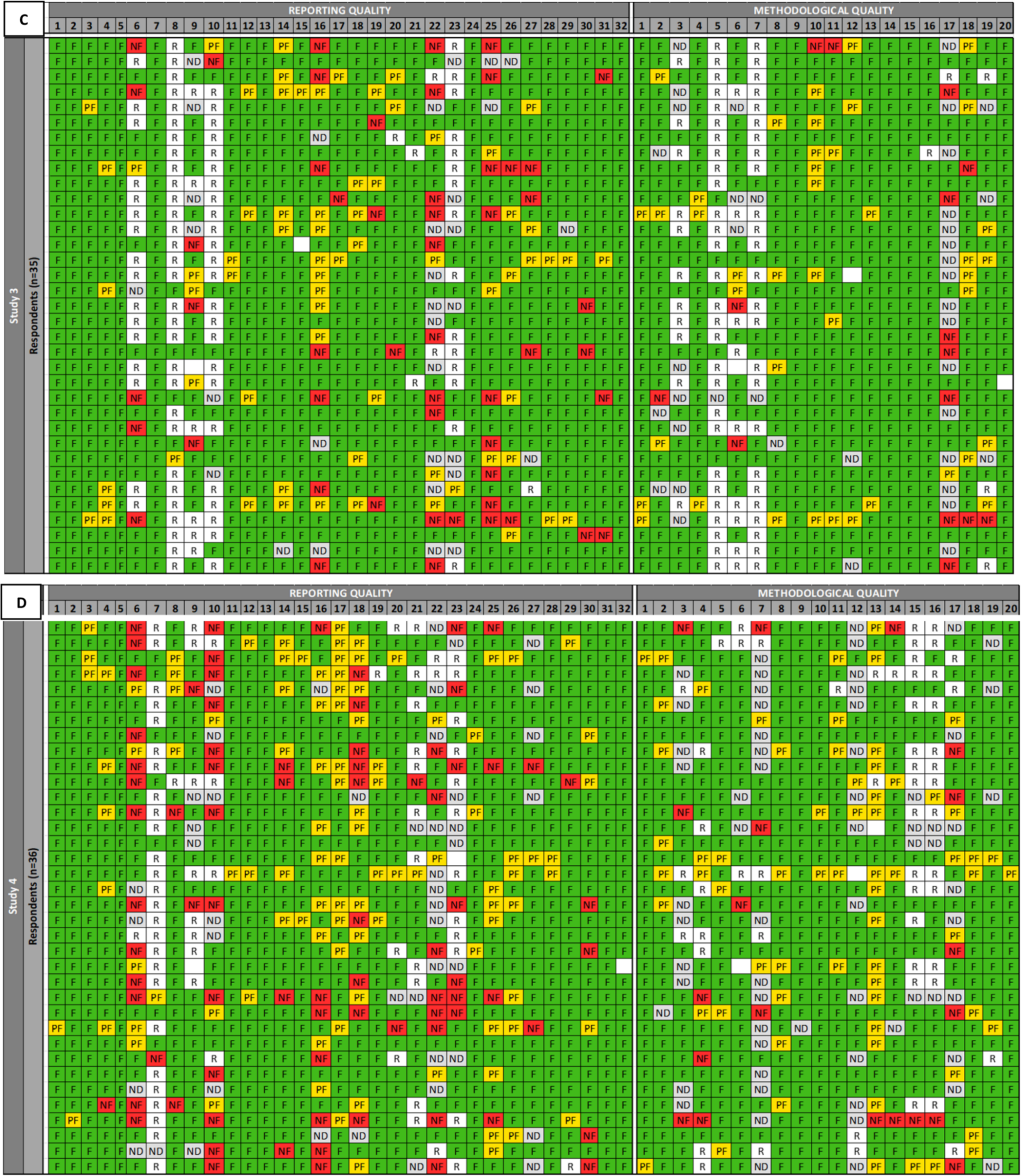
**A–D:** Details of the evaluations for study 1 (*n* = 35; Figure 3A), study 2 (*n* = 37; Figure 3B), study 3 (*n* = 35; Figure 3C), and study 4 (*n* = 36; Figure 3D) in the order of experts’ conclusions regarding reliability category. Each row corresponds to the evaluation by each expert; columns correspond to individual criteria. Green, yellow, red, gray, and white cells indicate criteria judged as fulfilled, partially fulfilled, not fulfilled, not determined, and removed from the evaluation, respectively.

In the SciRAPepi tool, version 1.0, the reliability section was divided into evaluating RQ and methodological quality (MQ) separately. In the RQ section, a higher degree of consistency, based on our decision rules that more than 50% of experts assigned the criterion to the same rating category, was observed for most criteria. However, some criteria, including criteria no. 6 (related to study participants and design), criteria no. 16, 22 (related to exposure and outcome measurement), and criteria no. 23 and 27 (related to data analysis) exhibited inconsistent evaluations, indicating the need for adjustments based on expert feedback from the online survey. Similarly, in the MQ section, while many criteria showed consistent expert evaluations, criteria such as no. 3 (related to study participants and design), 13, 16, 17 (related to exposure and outcome measurement), and 19 (related to data analysis) were identified to require further refinement. The comparison between the original and revised reliability criteria is provided in Table S5. Figure 4 shows the percentage of experts rating each study as “reliable without restrictions,” “reliable with restrictions,” “not reliable,” and “not assignable.” The most significant variation in expert evaluations was observed in Study 1, which may be attributed to its lower reporting and methodological quality compared with the other studies, leading to inconsistent evaluations. This is reflected in the reliability ratings from the online survey, where Study 1 was less frequently categorized as reliable with or without restrictions (74% of evaluations) compared to Study 2 (93%), Study 3 (97%), and Study 4 (93%). It is also important to consider that different evaluators may have different expertise and therefore may evaluate some criteria more strictly compared with others, which could contribute to perceived inconsistencies in the overall reliability assessment.

**Figure 4:**
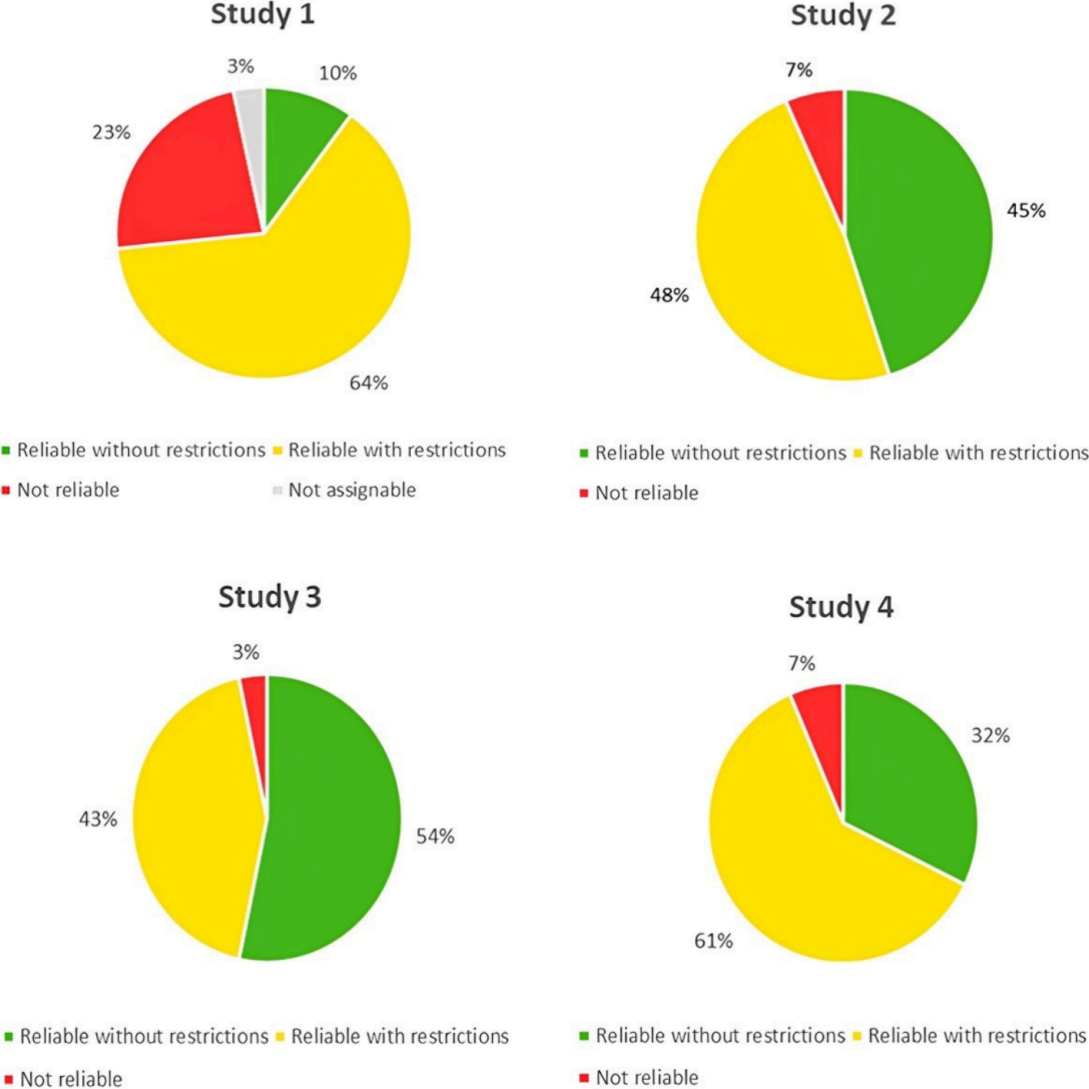
Distribution of how study 1 (*n* = 30 evaluations), study 2 (*n* = 31 evaluations), study 3 (*n* = 30 evaluations), and study 4 (*n* = 31evaluations) were categorized as reliable without restriction (green), reliable with restrictions (yellow), not reliable (red), and not assignable (gray), respectively.

Figure 5 shows the distribution of pooled criteria (fulfilled to not determined) within the studies judged as “reliable without restrictions”, “reliable with restrictions”, “not reliable”, and “not assignable”. Studies judged as “reliable without restrictions” and “reliable with restrictions” have a higher percentage of fulfilled criteria compared to studies in the rest of the categories. And, on the contrary, studies judged as “not reliable” or “not assignable” have a higher percentage of not fulfilled and not determined criteria compared to studies judged as reliable with/without restrictions. Overall, it can be assumed that there is a positive link between the number of fulfilled criteria and the reliability categorization of the study.

**Figure 5:**
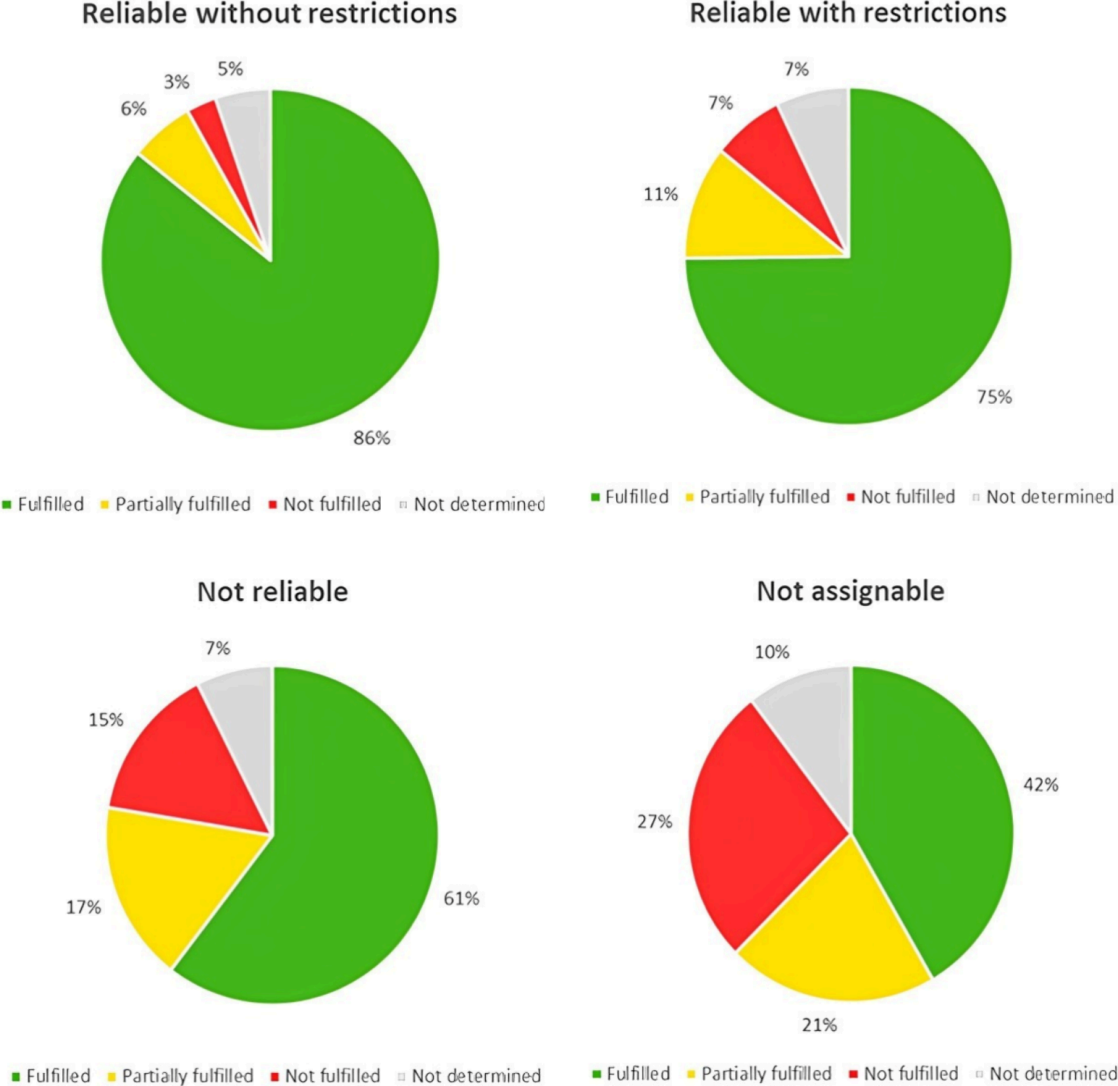
Percentage of pooled criteria judged as fulfilled (green), partially fulfilled (yellow), not fulfilled (red), and not determined (gray) in all evaluations resulting in categorization as reliable without restriction (*n* = 43 evaluations), reliable with restrictions (*n* = 66 evaluations), not reliable (*n* = 12 evaluations), and not assignable (*n* = 1 evaluation), respectively.

The same approach was applied in interpreting the results of the relevance section testing, revealing similar patterns shown in Table S5 and Figures S1–S3. Consistency in evaluations was observed for all relevance items, mainly no. 2, 3, and 4 (Table S5, Supporting Information Figure S1). Figure S2 presents the percentage of experts rating each study as “directly relevant,” “indirectly relevant,” or “not relevant.” The greatest variability in expert evaluations occurred in Study 1. The proportion of directly/indirectly/not relevant criteria in studies judged as directly/indirectly/not relevant. Although the relevance testing results followed a pattern similar to that of the study reliability assessment, it is important to note that specific research questions were not selected for determining the relevance of the studies. Therefore, these results primarily indicate whether the items are suitable for evaluating the relevance of epidemiological studies, but they were not intended for critically judging the relevance of the studies themselves.

When we compared evaluations based on the experts’ affiliation (academia, authority/government, industry/consultancy) and years of expertise in the epidemiology and risk assessment of chemicals, no clear pattern in evaluating the studies was observed.

### 3.2 Results of Online Survey

In the survey, experts were asked to evaluate the appropriateness of the RQ and MQ criteria as well as the relevance items. A significant majority of experts (97%) found the RQ and MQ criteria to be either appropriate or somewhat appropriate, while 88% of experts considered the relevance items to be appropriate or somewhat appropriate (Table 3). In addition, some of the experts noted that there was an overlap between the criteria from RQ and MQ. This suggestion was addressed in the SciRAPepi tool Version 2.0.

**Table 3:** Survey Regarding the Appropriateness of Criteria and Items in the SciRAPepi Tool

**appropriate criteria/items**	**reporting quality (RQ)**	**methodological quality (MQ)**	**relevance**
no	3.13%	3.13%	12.50%
somewhat	18.75%	21.88%	21.88%
yes	78.13%	75.00%	65.63%

Regarding the time spent evaluating each study, 56.25% of experts reported spending 1–2 h per study, 31.25% reported spending more than 2 h, and 12.50% spent less than 1 h. Notably, 90.63% of experts indicated that the time spent per study was reasonable (Figure 6).

**Figure 6:**
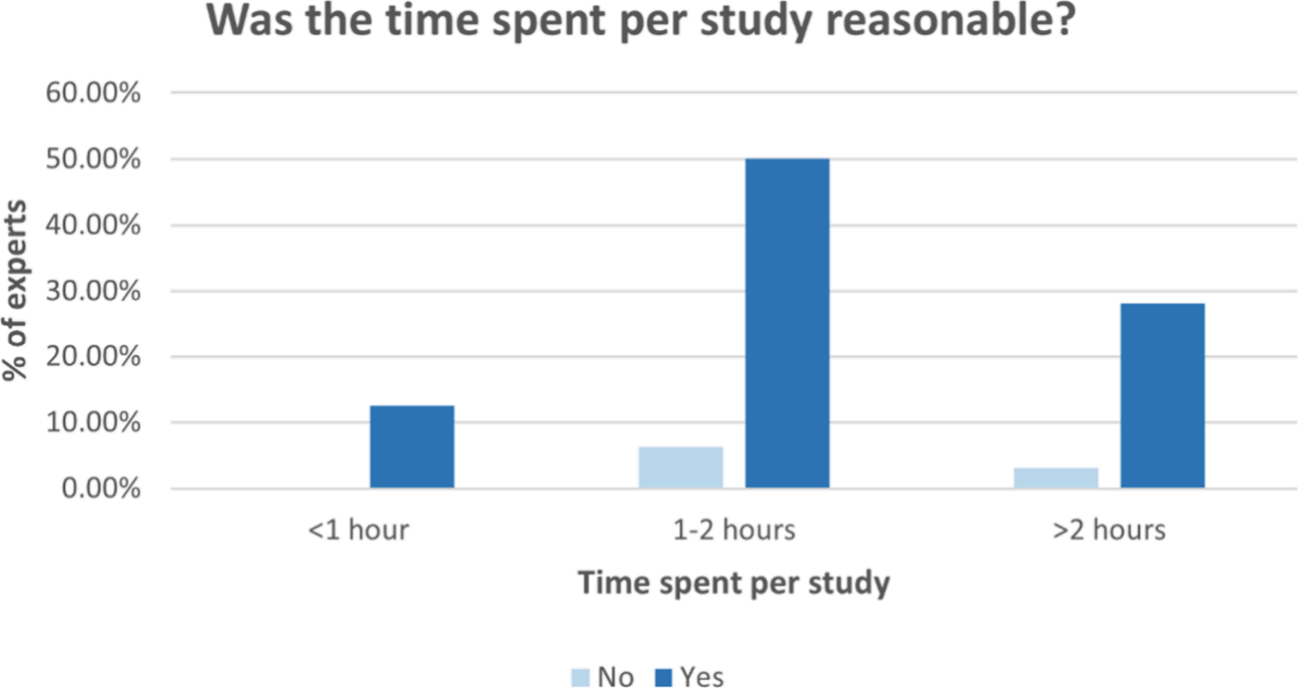
Survey regarding the time spent on evaluating studies.

The final section of the survey compared the SciRAPepi tool with other risk assessment tools across several dimensions: time required, user-friendliness, accuracy, and consistency of evaluations, the need for expertise, and the facilitation of transparency in evaluations (Figure 7). Experts were asked to rate each dimension as either better than, the same as, or not as good as the current approach. For clarity in graphical representation, responses of ‘do not know’ were excluded to emphasize the opinions of experts with direct experience in using risk assessment tools. The survey results indicate that SciRAPepi was generally regarded as a valuable tool, with experts rating it as equal to or better than other tools in most aspects. Over 50% of experts rated SciRAPepi as having a superior approach in all dimensions except for its ‘dependence on expert judgment and need for expertise.’

**Figure 7:**
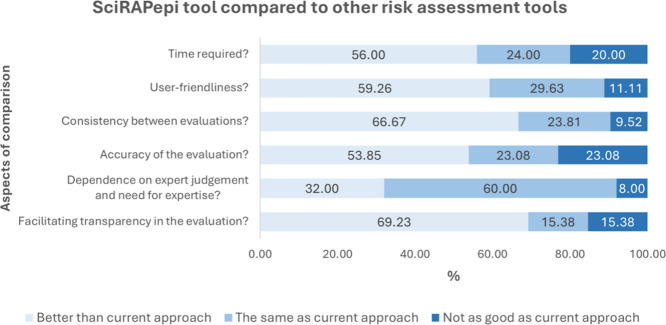
SciRAPepi tool compared with other risk assessment tools.

Experts were also invited to provide open-ended feedback on various aspects of the SciRAPepi tool. Common suggestions included:

- implementing a drop-down menu for study design that could automate choice of criteria for each study design,
- using criteria that integrate both exposure and outcome in relevant sections,
- sorting criteria by categories (e.g., exposure, outcome),
- reducing the use of terms associated with subjective opinions,
- including a dedicated space for comments,
- preventing errors when using macros,
- addressing redundancy between RQ and MQ criteria,
- offering more detailed guidance,
- providing a separate document containing all guidance items for easier reference,
- including examples in the guidance to enhance understanding,
- reducing the amount of text in the guidance to save time,
- clarifying the concept of generalizability to reduce confusion and subjectivity or removing this item from the tool,
- providing clearer criteria for evaluating relevance, especially in the absence of a research question.

This feedback highlights areas for potential refinement of the SciRAPepi tool.

### 3.3 Updating the SciRAPepi Tool

The results from the expert testing were used to develop a revised version of the SciRAPepi tool.

The SciRAPepi tool Version 2.0 includes several refinements such as

- combining criteria from reporting quality and methodological quality sections as “reliability”,
- removal of macros since they created technical issues,
- removing the function to increase weight of individual criteria; this function enabled users to increase weight of some criteria that they consider more critical or important for specific study design,
- change from interactive pie chart to bar chart visualizing the results of relevance,
- removal of relevance item - generalisability of the study,
- creation of separate sheets for different epidemiological study designs (cross-sectional, classical case-control, nested case-control, and cohort studies) and one comprehensive sheet containing all criteria and items,
- removing the CATEGORY column,
- using the term ″% fulfilled criteria” instead of “SciRAP score” in the results section,
- revision of the guidance for criteria that received inconsistent expert evaluations.

### 3.4 SciRAPepi Tool Version 2.0

The SciRAPepi Excel workbook consists of several sheets. Each sheet is dedicated to a specific epidemiological study design: cross-sectional, case-control, nested case-control, and cohort studies. There is also an additional sheet with all criteria that can be used for studies with a not clearly defined study design. Each sheet contains three parts: general instructions, reliability criteria, relevance items, and a result section.

Reliability criteria are grouped into six categories: participants, exposure measurement, outcome measurement, exposure and outcome measurement, analysis, and ethics/competing interests; and relevance items evaluate the appropriateness of study design, participants, exposure and outcome (bio)­markers, and analysis for a given risk assessment question. The tool allows assessors to score each reliability criterion as fulfilled, partially fulfilled, not fulfilled, not reported, or removed, while relevance items can be judged as relevant, indirectly relevant, or not relevant.

To support consistency between assessors, detailed guidance for each criterion and relevance item is embedded within the Excel workbook. The tool also includes an automated results visualization section that presents the proportion of “fulfilled,” “partially fulfilled,” “not fulfilled,” and “not reported” reliability criteria across different domains through bar charts. The bar chart for relevance items indicates the proportions of “directly relevant,” “indirectly relevant,” and “not relevant” items, respectively. Additionally, a summary table provides the overall percentage of fulfilled criteria, calculated using the formula:

$$\%\mathrm{fulfilled}\;\mathrm{criteria}=\frac{F+(\mathrm{PF}\times 0.5)}{T}\times 100$$ 1

where *F* = number of fulfilled criteria, PF = number of partially fulfilled criteria, and *T* = total number of criteria (excluding those removed).

A detailed description of all reliability criteria and relevance items, along with instructions on the use of the tool, is provided in Documents S1 and S2.

### 3.5 SciRAPepi Reporting Checklist

Based on the reliability criteria and relevance items developed in the SciRAPepi evaluation tool, a reporting checklist was developed. The reporting checklist is intended to support researchers in reporting study design, methods, and results in sufficient detail to facilitate data evaluation and use of epidemiological data in hazard and risk assessment of chemicals (Table S7).

## 4 Discussion

The aim of this study was to develop a SciRAP tool for the evaluation of the reliability and relevance of observational epidemiological studies for hazard and risk assessment of chemicals. The SciRAPepi tool provides structured and specific criteria to assess the reliability and relevance of epidemiological studies, facilitating their integration into regulatory decision-making.

While several tools exist to evaluate the quality of observational studies, each focuses on different aspects of the study evaluation. Together, they provide assessors with a broader range of options, allowing them to select the most suitable tool that aligns with their specific requirements and criteria when evaluating evidence for chemical hazard and risk assessment.^20–23^ The SciRAPepi tool, compared with other tools, is unique in that it integrates reliability and relevance assessment in one tool. It sets itself apart by integrating structured evaluation criteria with visual outputs and the % of fulfilled criteria, providing a clear and tailored method for assessing the reliability and relevance of epidemiological studies.

The SciRAPepi tool Version 2.0 includes several refinements based on expert feedback, including merging sections for RQ and MQ to the reliability section, removing macros and weighting option, removing generalizability item from relevance items, separating sheets per study design, and % fulfilled criteria emphasized instead of “score”.

The SciRAPepi tool complements the other Excel-based tools within the SciRAP platform, which includes tools for ecotoxicity, in vitro, and in vivo studies (www.scirap.org). To support efficient use, we developed detailed instructions and an Excel macro-enabled file that allows importing and visualizing multiple assessments in a single sheet.

The SciRAPepi tool is versatile and can be applied to a wide range of epidemiological study designs, including cross-sectional, classical case-control, nested case-control, and cohort studies. The exposure criteria are relevant for both biomonitoring and environmental exposure assessments (e.g., air, water, and soil), making the tool suitable for regulatory evaluations spanning human and environmental health. Assessors can remove nonapplicable criteria to align evaluations with specific study designs, but consistency in removed criteria is critical when comparing studies.

It should be noted that SciRAPepi, similar to the other SciRAP tools, aims to provide a qualitative assessment of reliability and relevance and does not include predetermined principles or cutoff values for categorizing the level of reliability and relevance of studies. Such approaches must be set up on a case-by-case basis and can, for example, entail identifying some of the SciRAP criteria as “key criteria” that must be fulfilled for the data set or study to be considered to be of sufficient reliability. The value for % fulfilled criteria can also be used together with the qualitative assessment, but must not be used on its own to categorize reliability or relevance. Some examples of how the output of the SciRAP evaluation has been used in WoE assessments and for categorizing reliability and relevance are provided in the published literature, for example, Ingre-Khans et al.,^27^ Holmer et al.,^28^ Röhl et al.,^29^ and Wiklund et al.^30^

The reliability criteria outlined in Document S1 and the reporting checklist (Table S7) may also serve as valuable resources for enhancing reliability and reproducibility during the peer-review process for scientific publications. This, in turn, could improve the use of such data in health risk assessment and regulatory decisions related to public health. Additionally, researchers can utilize the reporting checklist when preparing their manuscripts to ensure they meet the necessary standards to allow thorough evaluation of the reliability and relevance of their data and facilitate inclusion in regulatory assessments.

A strength of this study is the collection of data and feedback from participants with varying levels and types of expertise, spanning both epidemiology and risk assessment and representing academia, authorities, and industry. However, despite our efforts to engage researchers globally, by sending more than 300 invitations, we received data and feedback from 37 experts, primarily from Europe and the United States, limiting the generalizability of our results. The relatively low response rate is likely attributable to the considerable time commitment required to evaluate four different epidemiological studies. A follow-up reminder was also distributed to all potential participants; however, this did not substantially increase participation. In addition, due to the small group of participants and their uneven distribution across subgroups (e.g., affiliation), more detailed quantitative analyses were not statistically feasible in this testing round.

When comparing evaluations based on the experts’ affiliations (academia, government/authority, industry/consultancy) and years of expertise in epidemiology and risk assessment of chemicals, we observed no clear patterns in the evaluations. The consistency in evaluations regardless of the experts’ affiliations and areas of expertise suggests that the evaluation process may not heavily rely on the evaluator’s background. However, contrary to this observation, 68% of survey respondents believed that using the SciRAPepi tool relies significantly on expertise. This suggests expertise is crucial for evaluating studies with lower reliability, as exemplified by the variability in evaluations for Study 1. It is important to note that the aim of this tool is not to eliminate the need for expert judgment but rather to ensure transparency and structure in its application, while also harmonizing and standardizing the data assessment process to a certain extent.

Although we sought to refine the tool based on expert feedback obtained via an online survey, some recommendations require additional time and resources to implement. In the future, we aim to enhance the guidance by developing a separate, comprehensive document with detailed guidance and specific examples on how to evaluate each criterion. Concurrently, we plan to update the guidance provided in the Excel sheets, as some experts noted that the lengthy guidance is time-consuming when evaluating multiple studies. One suggestion involved creating an interactive Excel file where relevant criteria appear based on the selection of study design, thus minimizing confusion and reducing the evaluation time. While we have tailored SciRAPepi Version 2.0 by creating separate Excel sheets with criteria specific to different study designs, this proposed refinement is under consideration for future versions.

Additionally, some experts mentioned limited space for comments when using the tool. We have provided a designated “COMMENT” column in the Excel sheets for this purpose. While SciRAPepi is often compared to risk of bias tools, it is important to emphasize that it is not a risk of bias tool. We recognize that risk of bias tools typically allow for more extensive evaluator comments, as the criteria may require longer, more detailed responses or the criteria are phrased as open-ended questions. Our aim with SciRAPepi was to design a tool that covers the essential aspects of the study’s reliability through well-defined questions, reducing the need for lengthy explanations. Nonetheless, if any aspect of a study is unclear, evaluators can leave comments in the “COMMENT” column.

It is important to emphasize that the SciRAP platform and its tools are continuously evolving. Further updates and refinements to the SciRAPepi tool can be anticipated after it gains more use in the future. Since the tool was tested on a relatively small group of risk assessors and epidemiologists, it should be re-evaluated and updated after being applied in regulatory and academic settings over time. The SciRAPepi tool was developed to evaluate the reliability and relevance of observational epidemiological studies for use in chemical risk assessment. The expert feedback led to key refinements in SciRAPepi Version 2.0, improving usability and transparency. The tool shows potential for enhancing the reliability of epidemiological data in regulatory contexts. Future enhancements, including interactive features and more comprehensive guidance, will further streamline the evaluation process, enabling more efficient and accurate assessments.

## Supplementary Material



## Data Availability

Secondary data from expert testing and online surveys are provided within the manuscript or Supporting Information files. Anonymized raw data from expert testing (in the form of Excel assessment sheets) is available upon request. Raw data from online surveys is not publicly available to preserve individuals’ privacy under the European General Data Protection Regulation.

## Notes

SciRAPepi tool version 2.0 (Document S1), instructions for evaluating the reliability and relevance of epidemiological studies using the SciRAPepi tool (Document S2), details of the evaluations for study 1 (Figure S1), distribution of how study 1 (*n* = 30 evaluations), study 2 (*n* = 31 evaluations), study 3 (*n* = 30 evaluations), and study 4 (*n* = 31 evaluations) categorized in terms of relevance (Figure S2), percentage of pooled criteria judged as directly relevant, indirectly relevant, and not relevant (Figure S3), criteria to assess reporting quality of epidemiological studies, tool version 1.0 (Table S1), criteria to assess methodological quality of epidemiological studies, tool version 1.0 (Table S2), items to assess relevance of epidemiological studies, tool version 1.0 (Table S3), online survey, structured questionnaire (Table S4), results of the Expert Test Round Procedure (Table S5), comparison between the original and revised reliability criteria (Table S6), and SciRAPepi tool, Reporting checklist (Table S7) (ZIP)
