# Supplementary material for: SciRAPepi Tool: Optimization of the SciRAP Tool for Evaluating the Reliability and Relevance of Observational Epidemiological Studies for Hazard and Risk Assessment of Chemicals
Source: Environ Sci Technol. 2026 Jan 16;60(4):2952–63. doi: 10.1021/acs.est.5c11558 (PMC12874513; doi:10.1021/acs.est.5c11558)
Supplement: Supplementary file 1 [file es5c11558_si_001.zip › Supporting Information/Supporting Information/Supporting Information.docx]

***Supporting Information***

**SciRAPepi Tool: Optimisation of the SciRAP tool for evaluating the reliability and relevance of observational epidemiological studies for hazard and risk assessment of chemicals.**

Henrieta Hlisníková*^1^, Anna Beronius^1^

^1^ Institute of Environmental Medicine, Karolinska Institute, Nobels väg 13, 17177 Stockholm, Sweden

***Corresponding author:** Henrieta Hlisníková ([henrieta.hlisnikova@ki.se](mailto:henrieta.hlisnikova@ki.se))

**Contents**

[Document S1: The SciRAPepi tool version 2.0 3](#_Toc219360494)

[Document S2: Instructions for evaluating the reliability and relevance of epidemiological studies using the SciRAPepi tool 7](#_Toc219360495)

[Figure S1. Details of the evaluations for study 1 (n = 35), study 2 (n = 37), study 3 (n = 35), and study 4 (n=36) in the order of experts' conclusions regarding relevance category. Each row corresponds to the evaluation by one participant; columns correspond to individual criteria. Green, yellow and red cells indicate criteria judged as directly relevant, indirectly relevant and not relevant, respectively. 12](#_Toc219360496)

[Figure S2. Distribution of how study 1 (n = 30 evaluations), study 2 (n = 31 evaluations), study 3 (n = 30 evaluations) and study 4 (n = 31 evaluations) were categorized as directly relevant (green), indirectly relevant (yellow) and not relevant (red), respectively. 14](#_Toc219360497)

[Figure S3. Percentage of pooled criteria judged as directly relevant (green), indirectly relevant (yellow) and not relevant (red), in all evaluations resulting in categorization as directly relevant (n = 53 evaluations), indirectly relevant (n = 62 evaluations) and not relevant (n = 7 evaluations), respectively. 15](#_Toc219360498)

[Table S1. Criteria to assess reporting quality of epidemiological studies (tool version 1.0) 16](#_Toc219360499)

[Table S3. Items to assess relevance of epidemiological studies (tool version 1.0) 19](#_Toc219360500)

[Table S4. Online survey, structured questionnaire 20](#_Toc219360501)

[Table S5: Results of the Expert Test Round Procedure 23](#_Toc219360502)

[Table S6: Comparison between the original and revised reliability criteria 24](#_Toc219360503)

[Table S7: SciRAPepi tool - Reporting checklist 25](#_Toc219360504)

# **Document S1: The SciRAPepi tool version 2.0**

The SciRAPepi Excel workbook consists of several sheets. Each sheet is dedicated to specific epidemiological study design: cross-sectional, classical case-control, nested case-control, and cohort studies. There is also additional sheet with all criteria that can be used for studies with not clearly defined study design. Each sheet contains three parts as follows: general instructions, reliability criteria and relevance items, and a result section.

Reliability criteria (Table 1) are divided into several categories: participants (5-8 criteria depending on study design), exposure measurement (8 criteria), outcome measurement (8 criteria), exposure and outcome measurement (1 criterion), analysis (6 criteria), ethics and competing interests (2 criteria). The total number of reliability criteria is 34 for cohort studies. However, in case of cross-sectional and nested and classical case-control studies, the total number of reliability criteria is 31. There are 5 relevance items (Table 2) covering appropriateness of chosen study design, participants, exposure and outcome (bio)markers and analysis for specific risk assessment question.

Each reliability criterion can be assessed using a scale of "fulfilled", "partially fulfilled", "not fulfilled", "not reported", or "remove." Each relevance item can be categorised as "relevant", "indirectly relevant", or "not relevant", selected from a drop-down menu in the "selection" column of the Excel sheet. The "remove" option is available when a criterion is not suitable for a specific study. However, there is no option to remove for relevance items. The comment column in the SciRAPepi allows assessors to justify their evaluations.

**Table 1.** Reliability criteria in SciRAPepi tool version 2.0.

| **RELIABILITY** | |
| --- | --- |
| **Participants** | |
| 1 | The recruitment strategy and eligibility criteria for the participants were appropriate for the research question of the study. |
| 2 | The sample size was appropriate for the statistical analysis and study design. |
| 3 | The response rate of the potential participants was adequate. |
| 4 | The comparison/control group was appropriate for the research question and study design. |
| 5 | The follow-up of the participants was long enough to observe the outcome. |
| 6 | The outcome was absent in participants at the beginning of the study. |
| 7 | The loss to follow-up was ≤ 20% of the participants. |
| 8 | The baseline characteristics of participants were described (demographic, social, health status) in this study, or a reference was added if characteristics were described previously. |
| **Exposure** | |
| 9 | Reliable and sensitive methods were used for measuring the exposure (direct and indirect assessment methods). |
| 10 | The same methods were used for measuring the exposure in all participants. |
| 11 | Reliable (bio)matrices were used for measuring the exposure. |
| 12 | Chemicals (reference material, substances/chemicals used in the pre-analytical and analytical phases) and laboratory equipment used for analysis were of appropriate quality and purity to reliably measure the (bio)markers of exposure. |
| 13 | Reliable and specific (bio)markers were used for measuring the exposure. |
| 14 | Reliable laboratory test procedures (e.g., laboratories with external quality assessment scheme certificate - EQUAS certificate, using internal and/or external quality control samples) were used when measuring the exposure. |
| 15 | Appropriate percentage of samples was above the limit of detection (LOD) or limit of quantification (LOQ). |
| 16 | Samples were treated appropriately and with a low risk of contamination during the pre-analytical and analytical phases. |
| **Outcome** | |
| 17 | Reliable and sensitive methods were used for investigating the selected biomarker and/or outcome. |
| 18 | The same methods were used to measure the biomarker/outcome in all participants. |
| 19 | Reliable biomarkers were used for measuring the outcome. |
| 20 | Reliable biomatrices were used for measuring the outcome. |
| 21 | Chemicals (reference material, substances/chemicals used in the pre-analytical and analytical phases) and laboratory equipment used for analysis were of appropriate quality and purity. |
| 22 | Reliable laboratory test procedures (e.g., laboratories with external quality assessment scheme certificate - EQUAS certificate, using internal and/or external quality control samples) were used when measuring the outcome. |
| 23 | Appropriate number of samples was above the limit of detection (LOD) or limit of quantification (LOQ). |
| 24 | Samples were treated appropriately and with a low risk of contamination during the pre-analytical and analytical phases. |
| **Exposure & Outcome** | |
| 25 | The outcome assessors were blinded to the exposure assessment results, and exposure assessors were blinded to the outcome assessment. |
| **Analysis** | |
| 26 | Concentrations of biomarkers were matrix adjusted (if needed). |
| 27 | Appropriate data processing and statistical methods were used. |
| 28 | Important confounders and effect modifiers were identified. These important confounders and effect modifiers were appropriately accounted for in the study design or analysis. |
| 29 | Missing data were handled adequately in the dataset. |
| 30 | If applicable, results (significant and not significant) of the statistical analyses were presented in the form of levels of significance, size of the effects (e.g., correlation coefficient, β-value), and accuracy (e.g., confidence intervals). |
| 31 | Sensitivity analysis was performed to ensure the robustness of the results. |
| **Ethics and competing interests** | |
| 32 | The study was conducted with approval of Ethics Committee. Participants signed the informed consent at the beginning of the study. |
| 33 | The funding sources for the study were stated and all competing interests were disclosed (or it was explicitly stated that the authors had no competing interests). |
| **Other** | |
| 34 | Other aspects of study design, performance or reporting that influence reliability. |

**Table 2.** Relevance items in SciRAPepi tool version 2.0.

| **RELEVANCE** | |
| --- | --- |
| **Study design** | |
| 1 | The study design is relevant to the research question. |
| **Participants** | |
| 2 | The studied population group and the life stage of participants are relevant to investigate the research question. |
| **Exposure** | |
| 3 | The (bio)matrices used to measure exposure are relevant. |
| **Outcome** | |
| 4 | The studied outcome is appropriate for the risk assessment question. |
| **Analysis** | |
| 5 | The association between exposure and outcome is biologically relevant. |

As for previous SciRAP tools, guidance was developed for each criterion to ensure consistency among evaluators. This guidance was integrated into the SciRAPepi Assessment Excel workbook, which facilitates the evaluation process. It is incorporated into both, reliability and relevance sections, providing a comprehensive framework for evaluating epidemiological studies.

The results section of the SciRAPepi tool is included in the Excel workbook. The results are visually represented using bar charts, which illustrate individual groups of reliability criteria (Participants, Exposure, Exposure and Outcome, Outcome, Analysis, Ethics and competing interests), and items of relevance section (Study design, Population, Exposure, Outcome, and Analysis). The chart for reliability criteria uses green, yellow, red, dark grey colours to indicate the proportions of "fulfilled," "partially fulfilled," "not fulfilled," and “not reported” criteria, respectively. Removed criteria are not displayed in the chart. The chart for relevance items uses green, yellow and red colours to indicate the proportions of "directly relevant," "indirectly relevant," and "not relevant" items, respectively.

Additionally, the evaluation results in SciRAPepi are presented through one comprehensive table, showing the percentage of fulfilled criteria for reliability. Equation (1) shows the calculation of the % fulfilled criteria:

$\% fulfilled criteria=\frac{F+(PF\times0.5)}{T}\times100$

**(1)**

where 𝐹 is the number of fulfilled criteria, 𝑃𝐹 is the number of partially fulfilled criteria, and 𝑇 is the total number of criteria. Partially fulfilled criteria thus contribute half the value as fulfilled criteria. Criteria that have been removed are excluded from the calculation. The % fulfilled criteria can have a value ranging from 0% (all criteria are judged as "not fulfilled"/”not reported”) to 100% (all criteria are judged as "fulfilled”).

An instructions document was also prepared to explain how to properly use the SciRAPepi tool and interpret the evaluation results (Supplementary document S2).

# **Document S2: Instructions for evaluating the reliability and relevance of epidemiological studies using the SciRAPepi tool**

**Introduction:**

The SciRAPepi tool for evaluating epidemiological studies allows for evaluation of reliability and relevance. The evaluation often has to be endpoint-specific, meaning that the evaluation is carried out focusing on one of several endpoints investigated in the study. Separate evaluations may thus be necessary for different endpoints in one study. The evaluation may be conducted for either reliability, relevance, or both, depending on the purpose of the evaluation.

Download the excel files containing the assessment sheets available on the SciRAP website. Each excel file is tailored for specific epidemiological study design: cross-sectional, case-control, nested case-control, and cohort studies, as well as the file containing all criteria and items that are suitable for studies with no straightforward study design (**Fig. 1)**. Each sheet contain a brief introduction on using the SciRAPepi tool and pre-defined criteria/items to be evaluated in 2 sections for reliability and relevance.

**Fig. 1** Separate SciRAP epi Excel files tailored for specific epidemiological study designs.


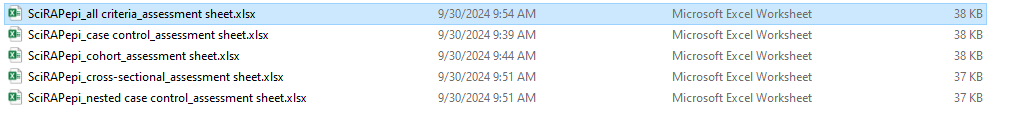


The reliability section is divided in specific categories: Participants, Exposure measurement, Outcome measurement, Exposure and Outcome measurements, Data analysis, Ethics and competing interests, and Other (**Fig. 2**).


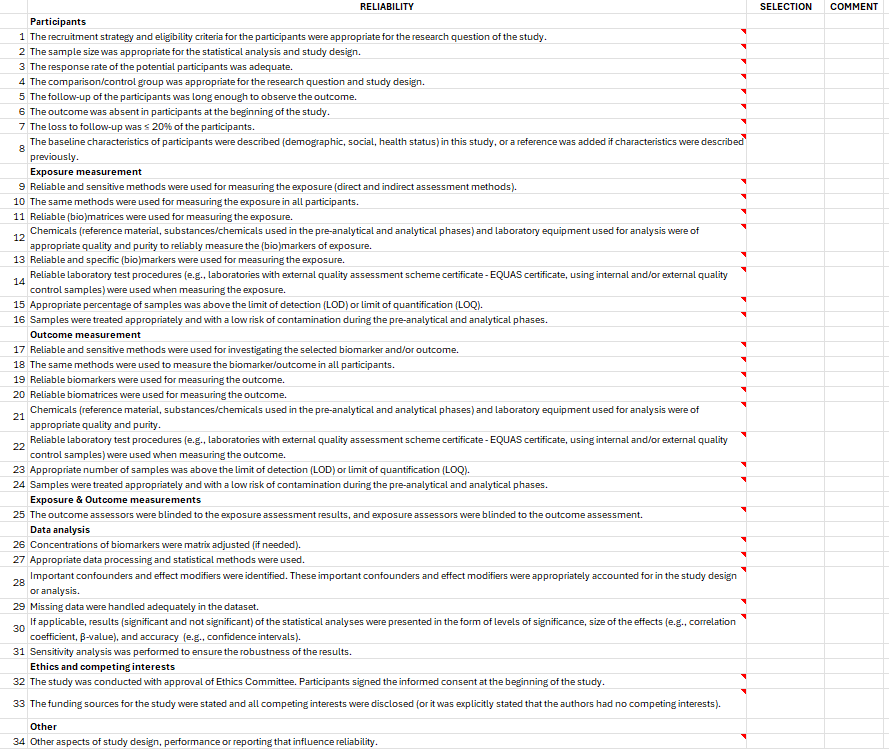


**Fig. 2** Categories of criteria in Reliability section of the SciRAPepi tool.

**Evaluation of the criteria:**

When you evaluate the criteria/items, choose one of the options from the drop-down menu in the "SELECTION" column (fulfilled, partially fulfilled, or not fulfilled for reliability section; directly relevant, indirectly relevant, or not relevant in the relevance section, (**Fig. 3**). This drop-down menu is in almost every cell in the "SELECTION" column.

**Fig. 3** Drop-down menu for the criteria in Reliability section of the SciRAPepi tool.


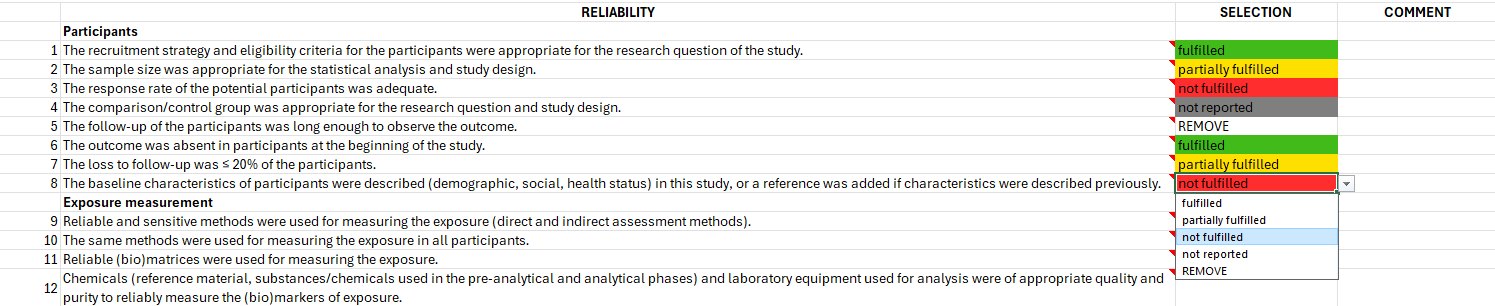


Guidance for evaluating individual reliability criteria and relevance items is available by pointing to the criterion with the cursor (the criterion containing the guidance has a red right corner, **Fig. 4**).


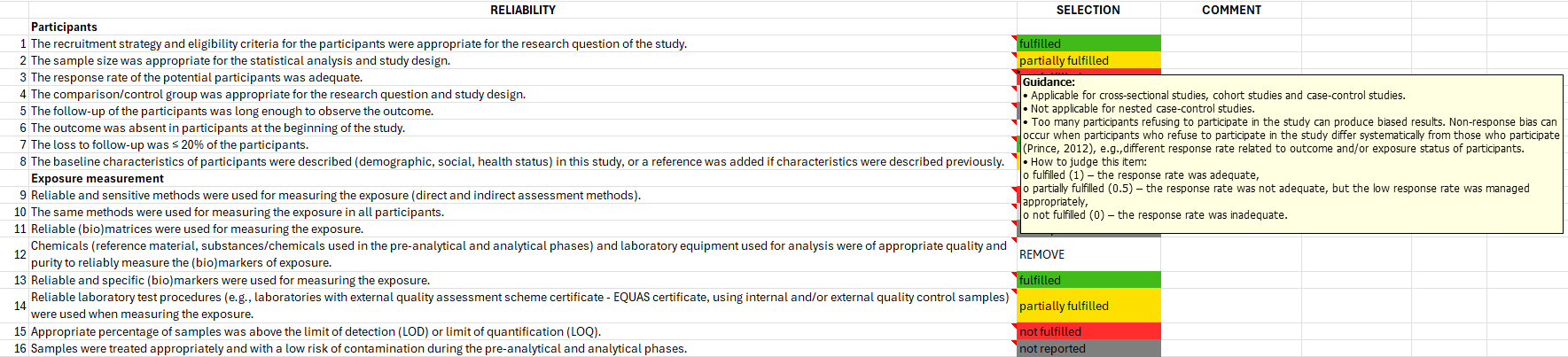


**Fig. 4** Guidance for evaluating each criterion in the SciRAPepi tool.

Criterion no. 31 (or 34 in “cohort” and “all criteria” Excel files) provides space for free text comments on additional aspects that affect study reliability. These criteria do not contain the drop-down menu with options.

You may use the "COMMENT" column to write free text comments, for example explaining your evaluation of a specific criterion (**Fig. 5**).


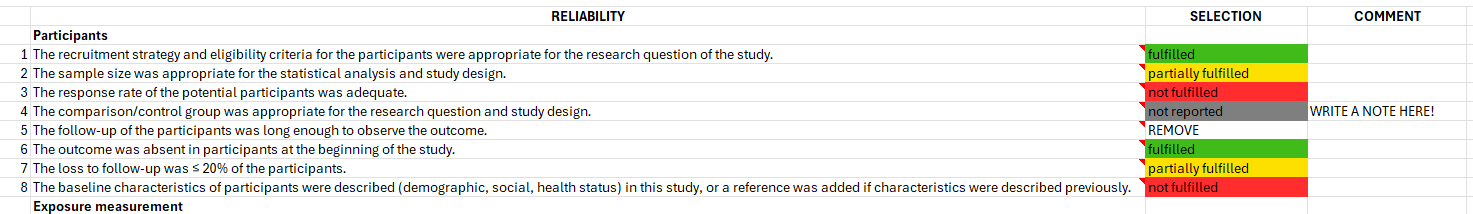


**Fig. 5** Writing a note in the "COMMENT" column.

***Judging criteria as “not reported”***

If a criterion cannot be judged, you can select the option “not reported” in the drop-down menu (**Fig. 3**). This might be used when sufficient information is lacking to make a judgment regarding whether the criterion is fulfilled or not.

***Removing criterion:***

Individual criteria may be considered more or less critical in the specific case you are working on, and the SciRAP tool includes a function to remove criteria for reliability. In that case, choose "REMOVE" in the drop-down menu of the "SELECTION" column instead of fulfilled, partially fulfilled, not fulfilled (**Fig. 3**). Removed criteria will not be included in the colour profile or % fulfilled criteria calculation. Motivations for removing criteria can be provided in the "COMMENT" column (**Fig. 5**).

NOTE: removing criteria will have an impact on the colour profile and the % fulfilled criteria. It is therefore important that the same criteria are removed in evaluations that are going to be compared to each other. Items in the Relevance section cannot be removed.

**Interpreting the results of the SciRAPepi tool:**

Results of the study assessment are shown right below the relevance section of the SciRAP tool in the form of % fulfilled criteria, as well as a colour profile.

**
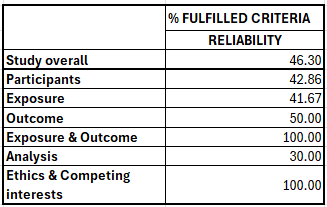
Fig. 6** Table with % fulfilled criteria.

***Percent fulfilled criteria***

The results show % fulfilled criteria of for the study overall, as well as for the specific criteria categories (**Fig. 6**).

- The % fulfilled criteria is calculated as follows:

$$\% fulfilled criteria= \frac{F+(PF*0.5)}{T}*100$$

where 𝐹 is the number of fulfilled criteria, 𝑃𝐹 is the number of partially fulfilled criteria, and 𝑇 is the total number of criteria. Partially fulfilled criteria contribute half the value as fulfilled criteria. Criteria that have been removed are excluded from the calculation.

The % fulfilled criteria can have a value ranging from 0 (all criteria are judged as "not fulfilled"/"not reported") to 100 (all criteria are judged as "fulfilled”).

NOTE:

- selecting “not reported” for a criterion will have the same impact as “not fulfilled” on the % fulfilled value. The user should take care to note the reason for leaving a criterion as "not reported".
- removing criteria will have an impact on the % fulfilled criteria, as well as the colour profile. It is therefore important that the same criteria are removed in evaluations that are going to be compared to each other.
- importantly, the % fulfilled criteria cannot be considered on its own but should be interpreted together with the colour profile when concluding on study reliability. The colour profile is crucial to identify where a study's strengths and weaknesses lie and is more informative than the % fulfilled criteria for this purpose.

***Colour profile***

In the colour profile, the evaluations of reliability and relevance are illustrated in bar charts (**Fig. 7**), showing green for fulfilled criteria, yellow for partially fulfilled and red for criteria that were not fulfilled. Criteria that were "not reported" will be shown as grey. Relevance items evaluated as relevant are shown as green, indirectly relevant items are shown as yellow, and if the item was evaluated as being not relevant for the risk assessment or problem formulation, it is shown as red. The bar charts do not include criteria that have been removed.

**Fig. 7** The evaluations of reliability and relevance are illustrated in bar charts.


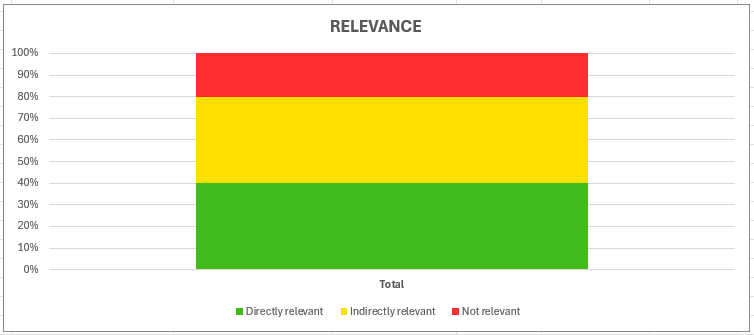

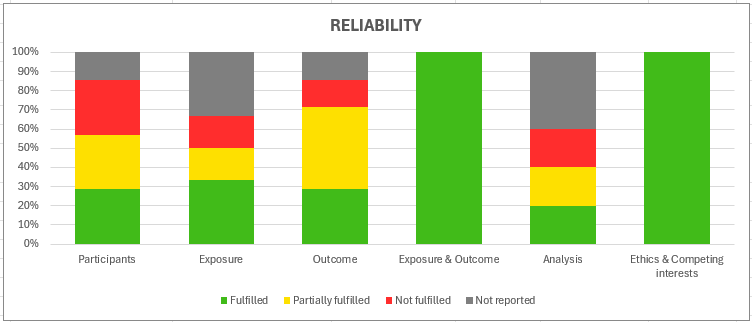


***Categorisation of reliability and relevance***

The SciRAP tool does not provide cut-off values or a pre-defined scheme for categorisation of the reliability and relevance of epidemiological data. Principles for such categorisation needs to be established on a case-by-case basis and should be fit for purpose for the assessment at hand.

**If you have any questions, please do not hesitate to contact us at** [**henrieta.hlisnikova@ki.se**](mailto:henrieta.hlisnikova@ki.se) **and** [**anna.beronius@ki.se**](mailto:anna.beronius@ki.se)**.**

# **Figure S1. Details of the evaluations for study 1 (n = 35), study 2 (n = 37), study 3 (n = 35), and study 4 (n=36) in the order of experts' conclusions regarding relevance category. Each row corresponds to the evaluation by one participant; columns correspond to individual criteria. Green, yellow and red cells indicate criteria judged as directly relevant, indirectly relevant and not relevant, respectively.**

# **Figure S2. Distribution of how study 1 (n = 30 evaluations), study 2 (n = 31 evaluations), study 3 (n = 30 evaluations) and study 4 (n = 31 evaluations) were categorized as directly relevant (green), indirectly relevant (yellow) and not relevant (red), respectively.**


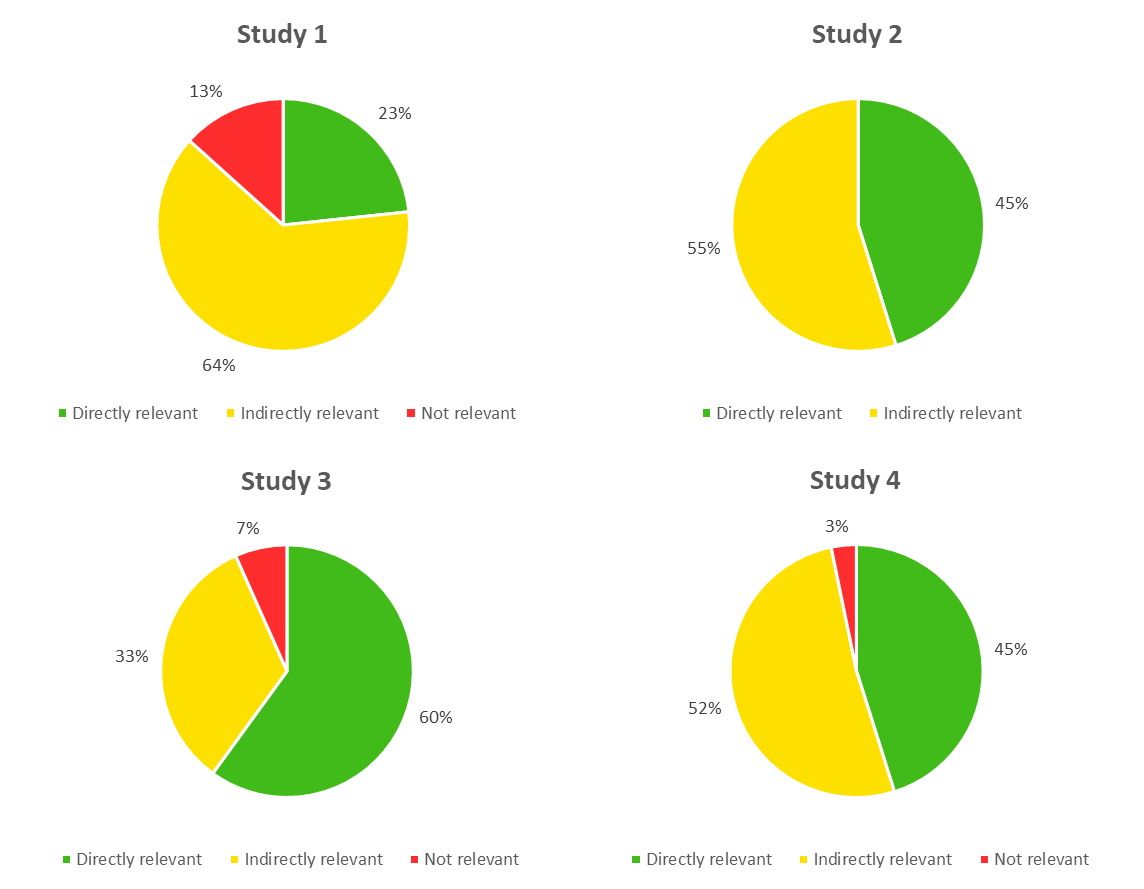


# **Figure S3. Percentage of pooled criteria judged as directly relevant (green), indirectly relevant (yellow) and not relevant (red), in all evaluations resulting in categorization as directly relevant (n = 53 evaluations), indirectly relevant (n = 62 evaluations) and not relevant (n = 7 evaluations), respectively.**


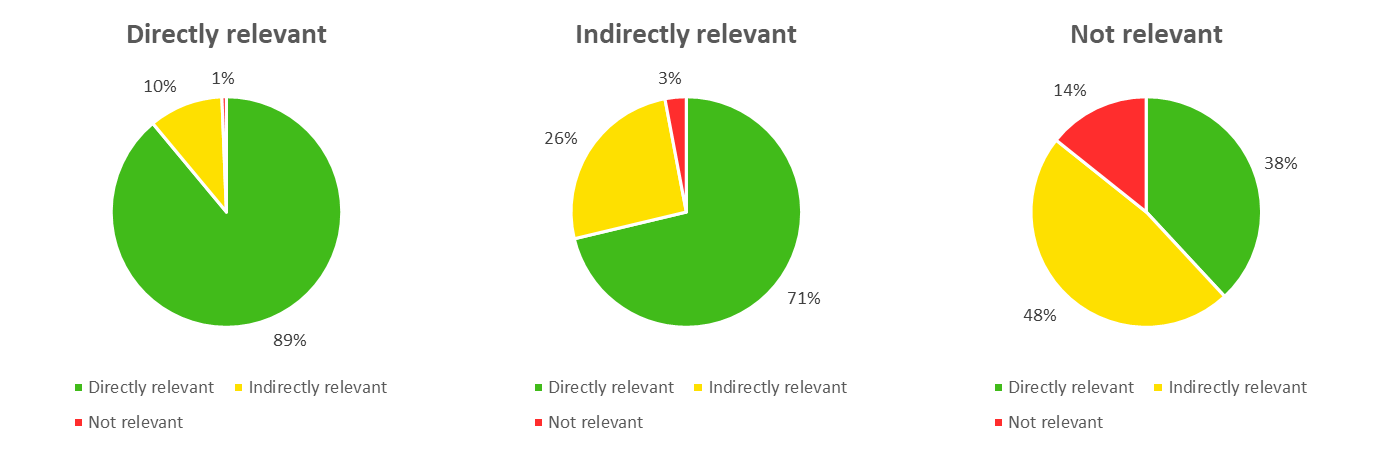


# **Table S1. Criteria to assess reporting quality of epidemiological studies (tool version 1.0)**

| **List of proposed criteria in the reporting quality section** | | |
| --- | --- | --- |
| *Criteria* | | *Category* |
| 1. | The study design was reported. | Participants |
| 2. | It was stated that all participants signed the informed consent at the beginning of their participation in the study. |  |
| 3. | All eligibility criteria for inclusion/exclusion of the participants to/from the study were clearly and sufficiently described. |  |
| 4. | The recruitment process was described precisely. |  |
| 5. | The number of participants was stated. |  |
| 6. | The response rate of the participants was stated in number or %. |  |
| 7. | The choice of the control group was stated. |  |
| 8. | The length of follow-up was stated. |  |
| 9. | The absence of the outcome of interest in the participants at the beginning of the study was stated. |  |
| 10. | The number and/or % of participants who withdrew their participation during the research were stated. |  |
| 11. | The baseline characteristics of participants were described (demographic, social, health status) in this study, or a reference was added if characteristics were described previously. | Exposure |
| 12. | The direct and indirect exposure measurement methods were clearly and sufficiently described in this study, or a reference was added if methods were described previously. |  |
| 13. | It was stated which (bio)matrices were used to determine the exposure and/or outcome. | Exposure/ Outcome/  Exposure+Outcome |
| 14. | The chemicals (reference material, substances/chemicals used in the pre-analytical and analytical phases), and equipment were described, or a reference was added if methods were described previously. |  |
| 15. | It was stated which (bio)markers were used to determine the exposure. | Exposure |
| 16. | The reliability of the laboratory test procedures was clearly stated (e.g., by external quality assessment scheme certificate - EQUAS certificate, using internal and/or external quality control samples). | Exposure/ Outcome/  Exposure+Outcome |
| 17. | The limits of detection (LOD)/limits of quantification (LOQ) for laboratory (bio)markers were stated, as well as the % of samples below/above the LOD/LOQ. |  |
| 18. | Sample treatment during the pre-analytical and analytical phases was described in this study, or a reference was added if the sample treatment was described previously. |  |
| 19. | The outcome measurement methods were clearly and sufficiently described in this study, or a reference was added if methods were described previously. | Exposure+Outcome |
| 20. | It was stated that the same methods were used for measuring the exposure and outcomes in all participants (e.g., from the control and case groups). | Exposure/ Outcome/  Exposure+Outcome |
| 21. | It was stated which biomarkers were used to determine the outcomes. | Outcome |
| 22. | It was stated that the outcome assessors were blinded to the exposure assessment results, and exposure assessors were blinded to the outcome assessment. | Exposure+Outcome |
| 23. | It was stated that the concentrations of biomarkers were matrix adjusted (if needed). | Analysis |
| 24. | The statistical methods and software used were described. |  |
| 25. | The method for identification of the potential confounders was stated. |  |
| 26. | All important confounders were correctly described. |  |
| 27. | The number of missing data for each variable used in the statistical analyses was stated. |  |
| 28. | The levels of significance and size of the effects (e.g., correlation coefficient, β-value) were stated when reporting results (if applicable for specific statistical test). |  |
| 29. | All results for the investigated exposure and outcome (not only the statistically significant ones) were reported as confounder-adjusted values, and their accuracy (e.g., confidence intervals) either in the body of the manuscript or in the supplementary material (if applicable for specific statistical test). |  |
| 30. | Ethics Committee approval was reported or a reference was added if it was described previously. | Ethics and competing interests |
| 31. | The funding sources for the study were stated. |  |
| 32. | Any competing interests were disclosed, or it was explicitly stated that the authors had no competing interests. |  |
| 33. | Was all information that is indispensable for evaluating the reliability of the data given? |  |

# **Table S3. Items to assess relevance of epidemiological studies (tool version 1.0)**

| **List of proposed items in the relevance section** | | |
| --- | --- | --- |
| *Items* | | *Category* |
| 1. | The study design is relevant to the research question. | Study design |
| 2. | The studied population group and the life stage of participants are relevant to investigate the research question. | Population |
| 3. | The (bio)matrices used to measure exposure are relevant. | Exposure |
| 4. | The studied outcome is appropriate for the risk assessment question. | Outcome |
| 5. | The association between exposure and outcome is biologically relevant. | Analysis |
| 6. | The results are generalisable for the external population. | Generalisability |

# **Table S4. Online survey, structured questionnaire**

| Personal information |
| --- |
| 1. I hereby give my consent that my personal data (name, e-mail, affiliation and years active in the field of research and/or risk assessment) is collected for the purpose of this study. I confirm that I have read and understand the information about handling of personal data in this study provided in the invitation to join the study. I understand that my participation is voluntary and that I am free to withdraw at any time without giving any reason. |
| 1. Your name |
| 1. What is your country of residence? |
| 1. What is your affiliation?  - Academia - Government/Authority - Industry - Consultancy - Other, please specify |
| 1. Name of employer: |
| 1. How many years’ experience do you have in the area of epidemiology?  - None - <1 - 1-5 - 5-10 - 10-15 - >15 |
| 1. How many years’ experience do you have in the area of risk assessment of chemicals?  - None - <1 - 1-5 - 5-10 - 10-15 - >15 |
| Study evaluation |
| 1. Based on your evaluation according to SciRAP, how would you categorise the reliability of Study 1/2/3/4? (*This question is repeated 4 times, once for each study*).  - Reliable – The study is very well reported, critical information about study design, conduct and results is included. Most SciRAP criteria for reporting quality were evaluated as “fulfilled” or “partially fulfilled”. All SciRAP criteria for methodological quality were evaluated as “fulfilled” or “partially fulfilled”. Single criteria may have been evaluated as “not fulfilled” or “not determined” if not judged to significantly influence the reliability of results or to bias results against the null. - Reliable with restrictions – The study is sufficiently well reported to allow for evaluation, i.e., the most critical information about study design, conduct and results is included. The study is generally well designed and performed but some minor flaws in the methodology may be present that could have influenced the results or biased the results against the null. - Not reliable – The study is sufficiently well reported to allow for evaluation, i.e., the most critical information about study design, conduct and results is included. OR the study has serious flaws in reporting, and it is not likely that gaining access to missing information will improve the reliability of the study. Critical SciRAP criteria for methodological quality were evaluated as “not fulfilled”. The study has clear flaws, primarily in how it was designed and performed, which are likely to significantly influence the reliability of results or to bias results against the null. - Not assignable – The study is too poorly reported to allow for evaluation; critical information about the study design or conduct that is needed to make an assessment of the study is missing. |
| 1. Based on your evaluation according to SciRAP, how would you categorise the relevance of Study 1/2/3/4? (*This question is repeated 4 times, once for each study*).  - Directly relevant - the choice of participants, (bio)matrices used to measure the exposure and biomarkers of outcome or outcome itself are relevant for measurement of human health effects or modes of key events related to human health effects. - Indirectly relevant - the choice of participants, (bio)matrices used to measure the exposure and biomarkers of outcome or outcome itself are partly relevant for measurement of human health effects or modes of key events related to human health effects. However, factors such as too specific population group or a smaller sample size reduce the relevance of the study. - Not relevant – the choice of participants, (bio)matrices used to measure the exposure and biomarkers of outcome or outcome itself are not relevant for measurement of human health effects or modes of key events related to human health effects. |
| The SciRAP approach |
| 1. How much time do you estimate you spent on average on evaluating each study using the SciRAP tool?    - <1 hour    - 1-2 hours    - >2 hours |
| 1. Is this amount of time reasonable?  - Yes - No - Comment: |
| 1. In your opinion, are the SciRAP criteria for evaluating reporting quality appropriate for the evaluation of epidemiological studies?  - Yes - Somewhat - No - Comment: |
| 1. Please specify any additional criteria that could be important for evaluation of reporting quality of epidemiological studies for health risk assessment. |
| 1. In your opinion, are the SciRAP criteria for evaluating methodological quality appropriate for the evaluation of epidemiological studies?  - Yes - Somewhat - No - Comment: |
| 1. Please specify any additional criteria that could be important for evaluation of methodological quality of epidemiological studies for health risk assessment. |
| 1. In your opinion, are the SciRAP criteria for evaluating relevance appropriate for the evaluation of epidemiological studies?  - Yes - Somewhat - No - Comment: |
| 1. Please specify any additional criteria that could be important for evaluation of relevance of epidemiological studies for health risk assessment. |
| 1. In the guidance for the last criterion of the relevance section ("Generalisability"), some of the considerations necessary for epidemiological studies to become relevant for the external population were mentioned. Are there any other considerations you would add to the guidance for the "Generalisability" criterion? |
| 1. Did you find the guidance provided with each criterion helpful?  - Yes - Somewhat - No - Comment: |
| 1. Did you find the offline colour-coding tool useful in the application of the SciRAP evaluation criteria for reporting, methodological quality, and relevance?  - Yes - Somewhat - No - Comment: |
| 1. Are you currently using any specific method for evaluating the quality and/or relevance of data for risk assessment? If yes, please specify which one(s) in the comments field.  - Yes, please specify which one(s) - No |
| 1. Compared to the approach for data evaluation you are currently using (even if no specific method is used), how does SciRAP compare concerning  - Facilitating transparency in the evaluation? - Dependence on expert judgment and need for expertise? - Accuracy of the evaluation? - Consistency between evaluations? - User-friendliness? - Time required?   Answer options: *Better than current approach*; *the same as current approach*; *Not as good as current approach*; *Don’t know* |
| 1. Please provide any other comments that could help further develop and improve the study evaluation criteria and offline tool in Excel. |

# **Table S5: Results of the Expert Test Round Procedure**

Available as a separate Excel file with four Excel sheets:

- Reporting quality,
- Methodological quality,
- Relevance,
- Selected criteria_improved

# **Table S6: Comparison between the original and revised reliability criteria**

Available as a separate Excel file.

# **Table S7: SciRAPepi tool - Reporting checklist**

| **SciRAPepi tool - Reporting checklist** | |
| --- | --- |
| Some of the items of this checklist were based on the STROBE combined checklist - STrengthening the Reporting of OBservational studies in Epidemiology, and Handbook for Conducting a Literature-Based Health Assessment Using OHAT Approach for Systematic Review and Evidence Integration. | |
|  |  |
| **Category** | **Items to be described** |
| **Purpose and aim** | Purpose and/or aim of the study. |
| **Outcomes** | Outcomes/biomarkers of outcomes included in the investigation. |
| **Ethical Statement** | Ethics committee approval. |
|  | Informed consent at the beginning of the study. |
| **Participants** | Description of study design. |
|  | Exclusion/inclusion criteria for the participants. |
|  | Sex of the participants. |
|  | Age and/or life stage of the participants. |
|  | Number of the participants. |
|  | Other baseline characteristics of the participants important for the study, e.g., nationality, ethnicity, race, education, socioeconomic status, place of residence, health status, consumer behaviour. |
|  | Response rate. |
|  | Recruitment of the participants. Follow-up of the participants. |
|  | Comparison/control group in the study. Matching controls with cases. |
| **Methods** | Methods should be described in enough detail to allow replication either in the Methods section or in another publication to which a clear reference is made. |
|  | Description of data collection. |
|  | Description of sample collection. |
|  | Description of the used laboratory equipment. |
|  | Description of the used chemicals during the pre-analytical and analytical stages. |
|  | Description of the used reference materials (standards) to analyze biomarkers. |
|  | Description of the used biomatrices. |
|  | Description of the measured biomarkers of exposure. |
|  | Description of the methods used for measuring the exposure. |
|  | Description of the measured outcomes/biomarkers of outcomes. |
|  | Description of the methods used for measuring the outcome. |
|  | Data supporting the reliability and sensitivity of the laboratory test method, e.g., participation in external quality assessment scheme -EQUAS, use of internal/external quality control samples, limits of detection (LODs) and/or quantification (LOQs). |
| **Statistics** | Details of statistical methods applied, e.g., data normalization, descriptive statistics, parametric tests, non-parametric tests, statistical modelling - descriptive models, explanatory models, predictive models. |
|  | Desciption of the methods for confounder identification. |
|  | Description of the handling the missing values in the dataset. |
|  | Desciption of the sensitivity analysis to determine robustness of the results. |
| **Results** | Number of participants in each step of the study. Number of missing values. |
|  | Descriptive data of the participants and their baseline characteristics. |
|  | Descriptive data of the exposure. |
|  | Descriptive data of the outcome. |
|  | Identified confounders should be reported. |
|  | All results for the association between exposure and outcome (not only the statistically significant ones) should be reported in the form of unadjusted and confounder-adjusted values and their accuracy (e.g., confidence intervals) either in the body of the manuscript, in the supplementary material, or available upon request. |
|  | Statistically significant results should be reported not only as "*p*-value" but also as the size of the effect, e.g., correlation coefficient, *β*-value. |
|  | Report of sensitivity analysis results. |
| **Discussion** | Summarization of the results with biological relevance. Comparing the results to other studies with the same or similar research question. |
|  | Explanation of the conflicting results (when comparing to other studies). |
|  | Limitations of the study. |
|  | Generalisability of the results for the external population. |
| **Other** | Source of funding. |
|  | Disclosure of any conflicts of interest. |
